# Promoting Obesity Prevention and Healthy Habits in Childhood: The OCARIoT Experience

**DOI:** 10.1109/JTEHM.2023.3261899

**Published:** 2023-03-27

**Authors:** Leire Bastida, Gloria Cea, Ana Moya, Alba Gallego, Eugenio Gaeta, Sara Sillaurren, Paulo Barbosa, Sabrina Souto, Eujessika Rodrigues, Macarena Torrego-Ellacuría, Andreas Triantafyllidis, Anastasios Alexiadis, Konstantinos Votis, Dimitrios Tzovaras, Cleilton Rocha, Lucas Alves, Pedro Maló, Márcio Mateus, Fernando Ferreira, María Teresa Arredondo

**Affiliations:** TECNALIABasque Research and Technology Alliance (BRTA) 48160 Derio Spain; Life Supporting Technologies-LifeSTechUniversidad Politécnica de Madrid 28040 Madrid Spain; Center for Strategic Health Technologies (NUTES) Campina Grande 44028-610 Brazil; Innovation UnitHospital Clínico San Carlos, IdISSC 28040 Madrid Spain; Information Technologies InstituteCentre for Research and Technology Hellas 57001 Thessaloniki Greece; Instituto Atlántico Fortaleza 60811-341 Brazil; NOVA School of Science and Technology 2829-516 Caparica Portugal; UNPARALLEL Innovation 8500-794 Faro Portugal; Coordenação de Formação e Inovação TecnológicaUniversidade de Fortaleza 28128 Fortaleza 60811-905 Brazil

**Keywords:** Children, health, healthy habits, Internet of Things (IoT), obesity prevention

## Abstract

Objective: Long term behavioural disturbances and interventions in healthy habits (mainly eating and physical activity) are the primary cause of childhood obesity. Current approaches for obesity prevention based on health information extraction lack the integration of multi-modal datasets and the provision of a dedicated Decision Support System (DSS) for health behaviour assessment and coaching of children. Methods: Continuous co-creation process has been applied in the frame of the Design Thinking Methodology, involving children, educators and healthcare professional in the whole process. Such considerations were used to derive the user needs and the technical requirements needed for the conception of the Internet of Things (IoT) platform based on microservices. Results: To promote the adoption of healthy habits and the prevention of the obesity onset for children (9-12 years old), the proposed solution empowers children -including families and educators- in taking control of their health by collecting and following-up real-time information about nutrition, physical activity data coming from IoT devices, and interconnecting healthcare professionals to provide a personalised coaching solution. The validation has two phases involving +400 children (control/intervention group), on four schools in three countries: Spain, Greece and Brazil. The prevalence of obesity decreased in 75.5% from baseline levels in the intervention group. The proposed solution created a positive impression and satisfaction from the technology acceptance perspective. Conclusions: Main findings confirm that this ecosystem can assess behaviours of children, motivating and guiding them towards achieving personal goals. Clinical and Translational Impact Statement—This study presents Early Research on the adoption of a smart childhood obesity caring solution adopting a multidisciplinary approach; it involves researchers from biomedical engineering, medicine, computer science, ethics and education. The solution has the potential to decrease the obesity rates in children aiming to impact to get a better global health.

## I. Introduction

Childhood obesity is a global paediatric public health concern in developed countries [1], [2], that affects around 224 million school-age children and is more prevalent among countries experiencing economic and nutrition transition [3]. Childhood obesity and overweight have been linked to societal problems such as easy access to inexpensive junk food and overexposure to junk food marketing; steadily increasing food portion sizes; decreased provision of healthy food choices and physical education in schools; lack of safety for outdoor activities in lower income areas; sedentary activities have become more popular such as watching television, playing videogames, and using a computer or mobile phone [4]. Several interventions have been developed over the years to treat childhood obesity, primarily including weight loss [5], [6]. Nevertheless, the best strategy to combat childhood obesity is to prevent the onset of obesity by encouraging healthy behaviours at an early age [7]. By using technology, such as smartphones and IoT devices, coupled with analytics capabilities [8], children can benefit from better and more personalised interventions tailored to their needs.

This paper presents OCARIoT, a solution designed to prevent obesity onset through the promotion of healthy habits among children aged 9–12 while solving the current limitations of similar systems [9], [10], [11]. The OCARIoT solution provides a dedicated DSS for health behaviour assessment and coaching of children and describes the other components of the IoT-based platform enabling the acquisition, integration, and training of multimodal datasets from IoT devices for environment and personal health monitoring. Overall implementation of the pilot program is presented, in which hypotheses were assessed to support the design of the intervention plan. Addresses a translational perspective on the results of this study through a multidisciplinary approach bringing together researchers from broad fields of expertise such as medicine with the participation of healthcare professionals, education enrolling schools and educators, biomedical engineering and computer science with technical profiles involved. The validation, evaluation and main findings are summarized as well.

## II. Methods and Procedures

### A. OCARIoT Project [12]

An H2020 EU project based on 12 institutions in Europe and Brazil that has developed a co-created experience to promote healthy habits acquisition and prevention of obesity in children (9-12 years old). The project provides an IoT-based personalized coaching platform for guiding children to adopt healthy eating and physical activity behaviours. An IoT network enables children to observe their daily activity patterns, health evolution, physiological and behavioural parameters, and environmental data. In combination with medical patterns, OCARIoT can tailor an interventional plan that allows children to remain active and engaged in their wellbeing and healthy habits following a gamification approach. OCARIoT has been deployed on three specific pilot sites in Brazil, Greece and Spain [13].

### B. Design Thinking Methodology

The co-creation process was established within the Design Thinking methodology [14] with the user at the centre of the system. This methodology was born in 70s, at Stanford University in California with the objective of providing solutions to users’ needs with technologies and business strategies that create added value for customers and suppliers.

**FIGURE 1.**
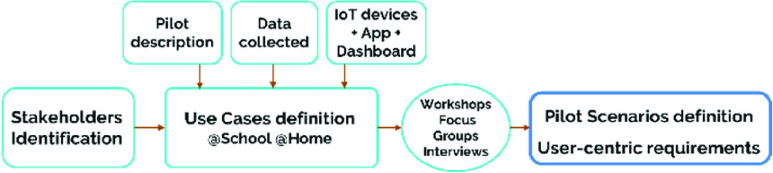
Design Thinking Methodology adapted to the OCARIoT context.

The adoption of a common definition of the user requirements and pilot scenarios was essential to identify the specifications for the OCARIoT solution including the exploration of involved profiles, existing problems or priority needs (Fig. 1).

### C. Microservice Reference Architecture

The microservice reference architecture presents two perspectives, the outer architecture and the inner architecture. The inner architecture is the implementation architecture of the microservices themselves, and the outer architecture delivers the platform capabilities to help all those little microservices and their DevOps teams work together to live up to the promise of flexible and scalable development and deployment [15].

## III. Results

### A. Stakeholders, Use Cases and Scenarios

Based on the established co-creation process, five stakeholders and their roles were identified: children, educator, family, healthcare professional and technology provider. Interviews, focus groups, Interactive Learning Cafés and workshops were selected as techniques to boost the participation on the validation and redefinition of the OCARIoT experience. A total of 8 co-creation sessions were performed in which 26 children, 11 parents, 12 educators, 8 researchers and 5 healthcare professionals participated. As results of these sessions, three use cases were determined with which a complete coverage is achieved in the relevant areas for the prevention of childhood obesity and the promotion of healthy habits: *1) Food tracking*, aiming to monitor the food frequency and food habits consumption of children; *2) Physical activity monitoring,* consists of recognizing what type of physical activity and how long children are active during the day. This functionality is enhanced by the addition of the IoT devices data collection such as wearables and smartwatches; *3) Motivational and educational activities*, aiming at long-term behavioural change of the children through educational activities and a gamified app which guides them to achieve their health goals in a funny and attractive way.

Gamification techniques (challenges, avatar, rewards, points, storytelling) were implemented to promote that children between 9–12 years old acquire healthy habits in the nutritional and physical activity areas while keeping them in long-term engagement using fun and easy mechanisms in the following scenarios: “@School”, key site where children are supervised by educators that push them to acquire the most relevant healthy habits; “@Home”, children interact with their relatives and the concepts learned should be strengthened. To enable a dynamic interaction among children and families/educators, the gamification strategy applied were designed by addressing extrinsic and intrinsic motivators (e.g., curiosity, acceptance, order).

### B. OCARIoT Solution

**FIGURE 2.**
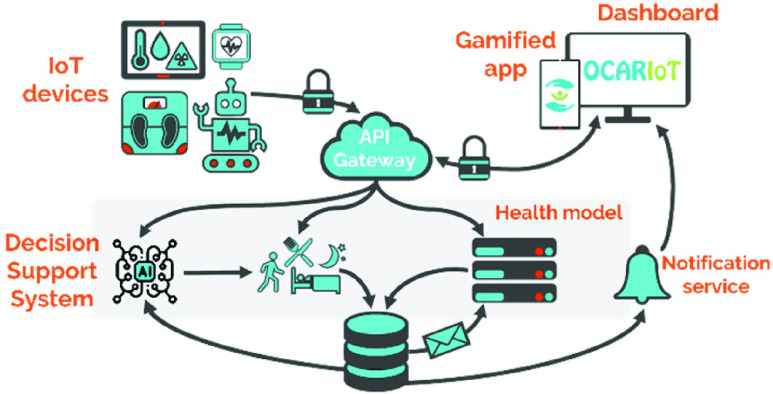
OCARIoT conceptual architecture.

Based on the specific requirements and use cases identified following the described user-centric approach, the OCARIoT architecture was defined. It follows the microservices paradigm, an architectural style which structures an application into a set of small, independently deployable services, as opposed to traditional monolithic approaches. It provides an Application Programming Interface (API) Gateway as the main entry point for initializing the microservices and their communication allowing the communication between clients (app, dashboard, embedded Gateway used by IoT devices) and microservices. Moreover, a notifications service provides real-time communication with push notifications. Fig. 2 depicts an overview of the conceptual components part of the architecture.

#### 1) Health Model

Integrates a set of variables, statistics and medical insights in order to identify the potential risk variables for obesity condition while analysing associations between different lifestyle habits -sleep duration and physical activity frequency- to create potential predictive models. The health model is used as basis for the design of the DSS and the integrated algorithms required for providing the personalised coaching plan. The health model is based on a set of variables, combination of statistical approaches and medical insights, creating the background for all the technological developments of questionnaires in the dashboard, in the gamified app for children, in the design of the DSS and in the IoT integration. The health model combines 2 different approaches validated after the final piloting finishes.

The first model approach demonstrates which variables are potential risk variables for obesity condition in the OCARIoT sample. The specific outcome is to identify obesity risk variables related to lifestyle habits that present association with obesity level, and to select which ones should be monitored in an intervention coaching plan through IoT devices. As a secondary outcome, the goal of the health model is to study the correlation between the anthropometric data and correspondent nutritional status categorization with the World Health Organisation (WHO) Body Mass Index (BMI)-for-age charts [16]. Moreover, the health model explores the relationship between novel variables like environmental ones and air pollution with the obesity categorization.

The second model approach establishes associations between different lifestyle habits to create potential predictive models for the behaviour of the children. The monitoring through the activity tracking/ fitness tracking IoT devices enable the study of the causal association and the analysis of time series of variables related to lifestyle habits, to detect variations over specific period of times (e.g., weekdays vs. weekends) or different months of the year (e.g., summer vs. winter). The association with the environmental variables oscillations is also included. The variables with a stronger association with sedentary behaviour are monitored and analysed within narrow time frames. The variables included in the OCARIoT health model are grouped into seven dimensions: 1) Socio-economic and demographic; 2) Health conditions; 3) Anthropometric; 4) Food intake and behaviour; 5) Physical activity; 6) Sleep habits; 7) Behaviour-psychological. Early risk variables that have presented association with obesity in the literature have been included [17], [18].

Instruments for data collection were adapted from previous epidemiological studies in Europe and Brazil: questionnaires for evaluating food intake and behaviour were based on PANCAKE [19] and ERICA projects, following the European Food Safety Authority guidelines and the EU Menu methodology [20]. The Physical Activity Questionnaire for Children (PAQ-C) [21] was included for intervention evaluation and validation of data from IoT devices.

The stratification for nutritional status was based on the BMI-for-age levels, the fat mass categorization [22] measured by skinfolds and by bioimpedance, and the abdominal obesity categorization [23]. The personalisation of the solution was based on an independent stratification for each set of variables included in the dimensions of diet, physical activity and education. Each variable has its own stratification in three risk levels, established by a combination between the evidence of the literature and the results of the baseline evaluation of the study developed in 2019. Target levels and the stratification for each variable configure the structure of the DSS. For the nutrition dimension, the cut-off points of the stratification levels are established with quantitative values corresponding to the frequencies of food consumption. For physical activity, the initial stratification is based on PAQ-C scoring, and adjusted by IoT inputs, with a mission/an intervention design based on dynamic percentage increases with respect to the individual baseline level until the target levels are reached. The outcomes from previous large systematic reviews were considered to design the intervention and coaching plan [24], [25], to promote behavioural change considering psychological factors and the specific environment and requirements of each pilot site.

#### 2) IoT Monitoring and Data Gathering

IoT devices enable the OCARIoT platform to monitor and collect data from children in a pervasive and continuous way, including different fitness wristbands (to measure physical activity and sleeping data) and smart weight scale (to measure weight and BMI). Furthermore, the platform collects environmental data of the schools running the pilots to increase the data available for the health model. To integrate information coming from the IoT devices into the OCARIoT platform, two components are provided: the Data Acquisition App (DAP) and the API Gateway. OCARIoT integrates Fitbit fitness wristbands, Fitbit Aria and Beurer smart weight scale. The information provided by these IoT devices is collected through the DAP and the IoT gateway. Furthermore, environmental and air pollution devices able to directly connect the API Gateway have been developed.

*Data Acquisition App (DAP) – Fitbit devices*

To integrate physical activity data coming from the IoT devices into the OCARIoT platform, the DAP implements two different approaches depending on the IoT device capabilities and connectivity constraints: (i) direct access to the device through a local communication protocol as Bluetooth Low Energy; (ii) third-party platforms integration (e.g. Fitbit) through an open REST API and asking for permission to access the data stored on their proprietary services. All the interactions are authenticated and authorized following the OAuth 2.0 Authorization framework.

*IoT Gateway – Smart weight scale*

The tracking of the children’s physical metrics requires multiple measurements of weight. To simplify the procedure, a smart weight scale system was developed, that automatically read the weight measurement from a smart scale and send them to the OCARIoT platform. The system is composed of 2 main elements: a smart weight scale, and a controller equipped with a keypad, a *Near Field Communication* (NFC) reader and a display. The controller reads the smart scale measurement through Bluetooth, associates it with the child ID being measured and send it to the platform using the available REST API. The child ID can be either specified using the keypad or identified through the ID registered in an NFC band.

The communications between the smart weight scale system are *Secure Sockets Layer* (SSL) connections. Each smart weight scale system authenticates against the platform using a scheme based the *Hyper Text Transfer Protocol Secure* (HTTPS) mutual authentication mechanism, where each device is configured with each own SSL certificate signed by a certificate authority managed by the OCARIoT platform. The smart weight scale system was designed to communicate with a smart weight scale available in the market to reduce costs, allowing schools to acquire smart weight scales at the best prices.

*IoT devices for measuring environmental data*

A significant part of the children’s activities takes place in the school environment. A couple of IoT systems were developed to obtain relevant environment data and automatise/facilitate the insertion of data into the OCARIoT platform. Those IoT systems collect data from the environment that could affect children’s physical activities and facilitate the collection of data that affects to the child’s weight and the insertion of the weight data into OCARIoT platform. Those IoT systems were designed to be low-cost, being the parts selected and assembled to create prototypes of low-cost products that can be acquired by schools with limited budget.

**FIGURE 3.**
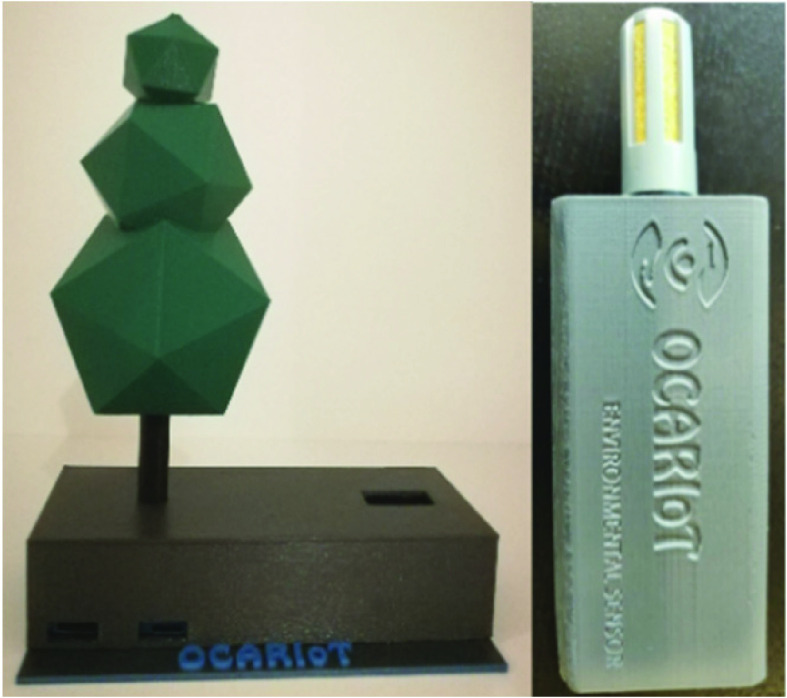
IoT environmental sensors.

An environmental sensor was developed to measure some environment parameters at schools. Air humidity and temperature impact on physical activity [26], [27]. The developed environmental sensor was designed to measure the air temperature and air humidity in places where children perform their physical activities. Such data that can be later correlated with the data from children activity to increase the accuracy about the children physical effort. While environmental data corresponding to outside activities can be collected from weather stations and services, indoor data must be collected using sensors deployed in that spaces. Fig. 3 (right) depicts the assembled sensor, mainly composed by 2 hardware elements: a processing board, and a humidity and air temperature sensor.

Other studies suggest that air pollution, including Particulate Matter (PM), is a potential factor that increases the risk of overweight and obesity [28], [29], [30]. An IoT device for measuring the levels of PM were incorporated in the pilot schools as illustrated in Fig. 3 (left). This device is designed with the ability to measure concentration levels PM1, PM2.5 and PM10, data storage in microSD memory and communication via WiFi with adaptability to communicate with the API Gateway. It also connects with multiple communication protocols and implements management skills in case of high levels of contamination, as was demonstrated in recent scientific publications [31].

#### 3) Decision Support System for Personalised Coaching Plan

Considering computerised decision support as the capability to generate useful knowledge and outcomes in regard to child’s health, behaviour, and progress through the use of computer-assisted tools, thereby supporting the decisions of children and their caregivers, and coaching them where required [32]. The health behaviour data is analysed by the DSS, which enables the provision of a personalised coaching plan for children in the form of missions (i.e. individualised goal-setting) as well as potential detection or prediction of behavioural risks. The DSS constitutes a back-end system dedicated for decision support and coaching, employing a rule-based approach for analysis of behavioural data, based on which the child’s self-reports and Fitbit data are processed according to specified rules in the dimensions of physical activity and sleep, diet, education/empowerment. The use of rules in the format of condition-action (IF-THEN) has been adopted in analysis of data according to international guidelines and expert knowledge, to provide a simple interface of communication between engineers and health/education professionals in the multi-disciplinary effort to define the operational logic of the DSS. Domain-specific rules are expected to be easily co-designed and understood by experts in nutrition, physical activity, and education, and they require limited development effort. Furthermore, rules have already proven to be effective in several medical engineering systems for health behaviour change [32], [33], [34]. The data analysis in the DSS enables the personalised mission selection (i.e. individualised goal-setting) for the children (*Mission Selection component*), the validation on whether the missions have been completed through feedback received by the carers (*Mission Validation* component), the triggering of motivation/reward messages to follow specific recommendations (*Motivation* component), as well as potential detection or prediction of behavioural risks (*Risk Detection/ Risk Prediction* components).

*Risk Detection/ Risk Prediction components*

The DSS supports health professionals in choosing a better coaching plan for children and when it should be applied; therefore, reference points are needed to indicate the start of the action. For that, a smart algorithm was created to indicate a valid reference point by analysing the trend of each health parameter (focused on physical activity and sleep data from smart bands), searching for moments in time (points) where those tendencies are increasing or decreasing steeply using the slope coefficient [35]. Thus, it is established a forecasting horizon to estimate the health variable state soon so that the coaching plans can be recommended regarding the current, past, and future states of the health parameters.

The smart algorithm is comprised of the next steps: 1) Insert the variables’ time series: A collection of time-dependent values; 2) Decompose the time-series and extract its trend; 3) Extract slopes from time-series’ trend using piecewise linear regression; 4) Check if the slopes are considered unhealthy following three types: positive or ascending, negative or descending and ignored when the slope is not deemed harmful (too steep); 5) If so, use the last valid detection as the reference point to commence forecasting and verify if the unhealthy behaviour continues in the predicted values; 6) In both cases of the trend being unhealthy, with unhealthy predicted values or unhealthy with non-unhealthy predicted values, these results has been considered by DSS when acting.

*Coaching plan generation*

**FIGURE 4.**
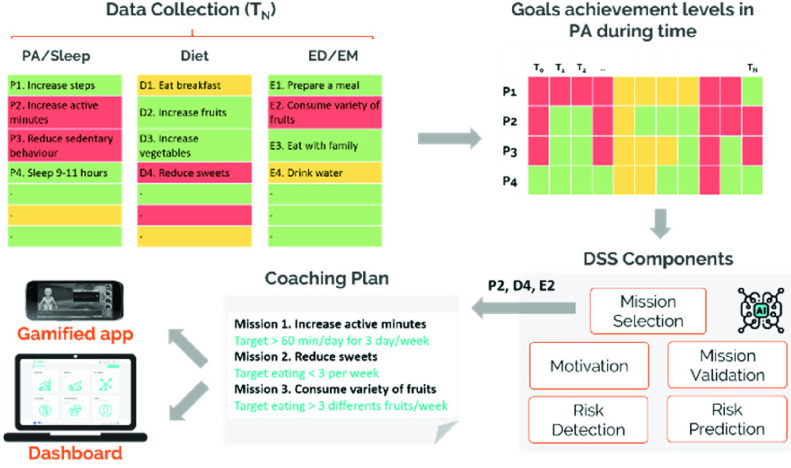
Coaching plan generation by the DSS (PA: Physical Activity, ED/EM: Education/Empowerment).

A central concept in the DSS architecture is the coaching plan which represents a set of prioritized personalised goals, i.e., missions, for the children according to collected and predicted behavioural data. Every week children and their careers are required to complete self-reports for behavioural data collection according to reliable instruments such as the Food Frequency Questionnaire (FFQ) [36] and the PAQ-C [21]. After processing the behavioural data (both Fitbit data and self-reports), the child is advised to follow one specific goal for each of the health behaviour dimensions of physical activity & sleep, diet, education & empowerment, for the following week. This is done according to a green/yellow/red flag decision scheme, according to responses provided in self-reports, in which a red flag denotes a behaviour which needs immediate change (i.e., prioritized behaviour), a yellow flag denotes a not optimal behaviour which requires change, and a green flag denoting an optimal health behaviour which needs maintenance. In the case two or more behaviours have the same flag, the history of most recent red flags is searched to prioritise the goal corresponding to the behaviour requiring more attention. Fig. 4 provides an overview of the data collection, behavioural assessment and mission selection for coaching plan generation according to green/yellow/red flag scheme. In this specific example, mission 1 targeting at the increase of active minutes is being selected, since behaviour P2 “Increase active minutes” is the behaviour remaining most recently, i.e., weeks $\text{T}_{\mathrm {N}}$, $\text{T}_{\mathrm {N-1}}$, $\text{T}_{\mathrm {N-2}}$, in red status.

The OCARIoT platform enables the communication of the DSS outcomes with the gamified app and the dashboard. This is possible because of the developed microservices used in the behavioural assessment, mission selection, mission validation or mission progress update.

#### 4) Dashboard for Families, Educators and Healthcare Professionals

**FIGURE 5.**
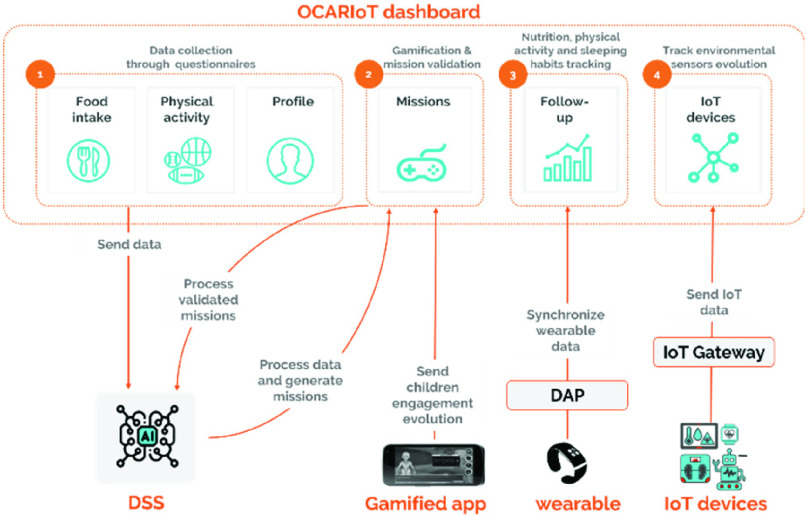
OCARIoT dashboard within the overall architecture.

Web-platform tool for families, educators and healthcare professionals to monitor children, consult the information reported by them through the gamified app, access tips and healthy lifestyle recommendations, monitor children’s evolution, visualize the collected data by IoT devices and also report additional information about heathy habits and food intaking. Concretely, families and educators are responsible for encouraging, tracking and helping children to adopt healthy habits, while the healthcare professionals can follow up and analyse the variables collected and provide specific health interventions and recommendations. Fig. 5 illustrates how the dashboard interacts with other architecture components providing support in (1) data collection, (2) gamification and mission validation, (3) daily habits tracking and (4) environmental sensors tracking.

*Data collection through questionnaires*

Making use of standardized questionnaires, such as *Aladino* [37] used for clinical studies on childhood obesity, different children’s parameters are collected to know: Socioeconomic and demographic characteristics of the participant’s parents or guardians; Children’s and parents’ health profiles; Frequency at which parents and children practice physical activities; Children’s diet and eating habits; Sleeping habits; The feelings of the children during food intake and the duration of meals; Aspects related to the child’s perception of his or her own body. Besides these data, the dashboard allows introducing the children’s anthropometric measurements to monitor and detect their nutritional status.

*Gamification and mission validation*

It represents a key point in the whole system being the crossroad between the dashboard, the DSS and the gamified app. It integrates the user engagement report coming from both, the gamified app and the dashboard, and the missions generated by the DSS, aimed at improving children’s nutritional, physical activity and educational habits. Thus, the dashboard provides a complete overview of the users’ engagement status collecting the points and the percentage of the dexterity, discipline and intelligence abilities obtained completing missions and games in the app. Moreover, in this part of the dashboard users can visualize the three weekly active missions with their respective recommendations as well as report the achievement of them. Finally, it contains the agent report questionnaire, which gives some complementary information to the DSS for the generation of new missions.

*Nutrition, physical activity and sleeping habits tracking*

An overview of the children’s behaviours and daily activity patterns in relation to nutritional habits, physical activities and sleeping habits. The physical activity and sleeping habits parameters collected by the children’s wearables are imported and presented in a bar chart. The most updated wearable measurements are only shown when the synchronization of the data through the DAP application is performed. Finally, users can consult the food registers added manually by parents and educators as part of the *data collection* functionality.

*Track environmental sensors evolution*

The evolution of environmental parameters that influence obesity such as Air Quality Index (AQI) [38], humidity and temperature are tracked. This information is collected by an embedded gateway and then integrated and presented in the IoT devices screen making use of different line charts.

#### 5) Gamified App for Children

**FIGURE 6.**
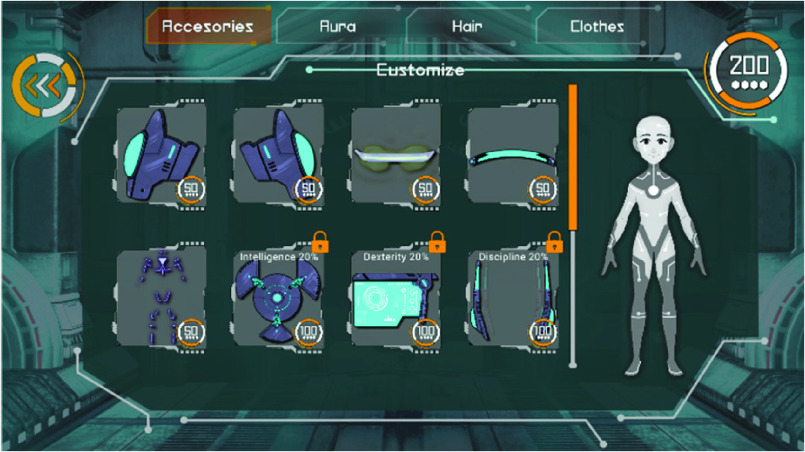
OCARIoT App: mentor personalisation.

The goal of the OCARIoT App is to motivate and make fun the acquisition of knowledge and the application of healthy habits in daily life, in addition to promoting autonomy of children and collaboration within the family and educators. It has been developed specifically for children drives their engagement and behavioural change while having a clear focus on preventing obesity. The User Interface has followed the aesthetics of this agent secret storytelling, challenging children with a set of missions to be completed in the real-world about healthy habits focused on physical activity, food intake and knowledge acquisition. The expected results aimed to be achieved by children are: embracing healthy habits; acquisition of knowledge about healthy habits and understand how to apply them in their daily life; making the learning process about healthy habits as memorable as possible while using the app. Four core elements are integrated in the app to support the gamified experience [13]:

• Considering the requirements collected from the pilot sites and the preferences of children, an agent storytelling was selected, where the children must develop three abilities through the completion of a set of missions for becoming an agent. Missions can be individual, if performed by the child alone, or social, if they are performed together with family or at school.
• The mentor represents the person who challenges children with missions while providing guidance during the whole experience. It provides the tutorial, with the rules, the objective, the rewards and all the things the child should know to go inside the experience and back again. The mentor can be personalized by using the points gained when completed missions (see Fig. 6).
• The educational games give the child the ability (knowledge) to overcome a mission, i.e., actions to carry out in real life. The four types of educational games have been integrated in the app: “Trivia”, “True or False”, “Matching pairs” and “Find them all”. By playing these educational games, the child also wins points.
• The Agent report provides children with the status about the abilities progress, the number of missions achieved, and the total points acquired. Additionally, once a week the children must submit a report by answering 6 questions related to their behaviours during previous week about food intake and physical activity. This information is being used by DSS for the personalization.

The OCARIoT App interacts with the rest of the architecture components providing support in (1) data collection through the agent report, (2) list of available mission to be completed provided by the DSS and (3) list of missions pending for validation. It is provided as an Android application and as a webapp to easy the access for children.

#### 6) Testing and Bug Tracking

The whole IoT platform has followed a validation process [39], [40] to ensure that the software meets and fulfils all the functional requirements. Validation process for testing the functionalities of the overall OCARIoT has been applied for each component separately including basic tasks to define how the tests were elaborated, performed, and analysed. Then, it was performed end-to-end tests, to check the data correspondence between front-end and back-end. Moreover, the integration among the all components was tested and the real users’ scenarios were simulated by using some Fitbit fitness wristbands to collect test users’ data.

### C. Piloting Results

The piloting is developed in two consecutive phases with specific outcomes: in phase 1 the validation of low fidelity prototypes while in phase 2 the assessment analysis and intervention evaluation in real settings. The data collected during 2018–2019 school year was used for training obesity modelling and algorithms. The pilot sample in phase 2 includes intervention and control group. For the calculation of the sample size by hypothesis contrast technique the main outcome considered was the decrease in obesity prevalence: significant statistical results to extrapolate that the childhood obesity rate is decreased (at least 50%) when the proposed solution is used after the project lifetime; at least 90% of children’s BMI are in the healthy range. This implies to achieve at the end of the project a prevalence of overweight or obesity of 10% of the whole sample. Considering the real prevalence of obesity globally in the sample and a 12% of expected proportion of losses, the sample size per group is 75 children, achieved globally in the 3 countries. The cohort included in phase 1 piloting was 334 children recruited in 4 primary schools located in Greece, Spain, and Brazil. Baseline results presented in [41] are summarised in Table 2.1

**TABLE 1:** Gamified App Ability and Healthy Habits Relation

Ability	Relation with healthy habits
Dexterity	Physical activity: agility, power, endurance, resistance to overcome the tests.
Intelligence	Teach children all the aspects related to the healthy habits that will be put in practice with the challenges.
Discipline	Provide children awareness and self-control over the impulses for eating, actions related to sleeping patterns, and to teach them to make better food choices.

**TABLE 2:** Descriptive Results From Phase 1 Piloting

	Global	Spain	Greece	Brazil
Subjects, n	334	60	156	118
Female (%)	58.7	56.7	59.6	58.5
Age (years)	10.26 ± 1.36	10.63 ± 0.96	10.12 ± 1.45	10.09 ± 0.83
obesity (%)	11.7	8.3	5.8	21.2
overweight (%)	17.1	11.7	17.9	18.6
normal weight (%)	65.3	75.0	69.9	54.2
thinness (%)	5.1	5.0	4.5	5.9
severe thinness (%)	0.9	0,0	1,9	0,0
PAQ-C scoring	2.75 ± 0.56	3.04 ± 0.60	2.63 ± 0.50	2.78 ± 0.56
Sleeping hours	9.16 ± 1.21	9.75 ± 1.14	8.93 ± 1.32	9.21 ± 1.12

Regarding gender distribution, 58.7% were female. Mean age was 10.26 years (±1.358). The mean global prevalence of obesity and overweight globally was 11.7% and 17.1% respectively, which equates to a 28.8% of children with a non-healthy BMI status. According to recent data from the WHO, this prevalence is similar to the data collected in the Childhood Obesity Surveillance Initiative (COSI) survey [42], where 1 of every 3 children of 11 years of age presents overweight or obesity. Brazil presented the higher prevalence of obesity (21.2%) and the second position in overweight (16%) prevalence while Greece presented the lowest prevalence of obesity, with a mean value of 5.8%. Globally, the percentage of children with high and very high levels of adiposity was 13.9% and 8.9% respectively.

There is a significant variability between countries, Brazil has the highest rates of high and very high fat level, nearly the double of the mean global value (22.9% and 16.5% respectively). Abdominal obesity was analysed in a subgroup of 175 patients with waist circumference measurement, with a 30.5% prevalence. The value was similar to the global value of overweight and obesity according to BMI. Potential obesity risk variables were identified in phase 1 piloting associated with the BMI-for-age categorization, in the model approach 1 and 2 deployed by partners. The set of analysis deployed gave the basic outputs to design the intervention plan together with the validation and evaluation.

• The variables related to the association of fat mass values measured by skinfolds ( $\text{p}< 0.001$) and bioimpedance ( $\text{p}< 0.001$), and waist-to hip-ratio (p=0.009) -with obesity condition by BMI categorization in the 5 levels of nutritional status [16], were included in the final evaluation purpose for potential use of IoT devices. This enables the comparison of the evolution of the different anthropometric variables that could be considered to monitor the “obesity condition” as a criterion variable, apart from the BMI-for-age categorization. Moreover, the inclusion of new Key Performance Indicators (KPIs) based on novel anthropometric variables in future interventions for validation purpose.
• The strong association of weight at birth ( $\chi ^{2}=2.48$; p=0.015) and parental BMI, both from mother ( $\chi ^{2}=9.01$; p=0.029) and father ( $\chi ^{2}=15.68$; p=0.001), with obesity and overweight condition confirm that the early risk variables that presented association with obesity in the literature [17], [18], were also replicated in Phase 1 data.
• The variables from food dimension associated with nutritional status based on BMI categorization in 5 levels or abdominal obesity categorization were sweets ( $\chi ^{2}=46.1$; p=0.017), sugary sodas ( $\chi ^{2}=41.43$; p=0.049) and pizza consumption ( $\chi ^{2}=7.72$; p=0.021). The variables related to sedentary behaviour that had association with obesity categorization were screen time during meals ( $\chi ^{2}=18.1$; p=0.02), time with smartphone ( $\chi ^{2}=9.92$; p=0.002). Having hypercholesterolemia was also associated with obesity condition ( $\chi ^{2}=7.81$; p=0.005). There was a significant association between practising sports with parents and abdominal obesity ( $\chi ^{2}=5.4$; p=0.02), with a rate of healthy children of 63.64% in the group of regular practising family activities.
• Hypothesis test revealed that lack of sleep (below recommended hours for age) was associated with a reduced Moderate to Vigorous Physical Activity (MVPA) level in the following day. MVPA and sleeping hours are the pillars of the physical activity intervention through the continuous monitoring by IoT devices.

The results obtained in the Phase 1 have given the insights for mission selection and prioritisation, personalization of the variables included in each dimension and rules to be included in the DSS. The food groups with significant associations have been included in the DSS with a higher leadership in the coaching plan, with additional missions regarding food recipes and practical material to be delivered to families.

In Phase 2, 127 children completed the final evaluation, stratified by intervention group (77.9%) and control group (53%) in the four schools from Spain, Greece and Brazil. After the 5 months of the piloting phase, results revealed that 74% of the proposed missions were completed by children, improving their health behaviours. In the intervention group, consumption of the recommended intake of fruit, more than 2 pieces of fruit a day, was achieved by 42,62% compared to the initial 7,69%. Also, the vegetables consumption increased from 21,79% to 59,02%. In the same line, it is important to highlight the identification of potential variables related to the dimensions of the healthy model that present a trend association with a non-healthy nutritional status. The main insights were higher consumption of bread toasts, cold cuts, yogurt and bakery products; and lower consumption of cheese. Moreover, a higher consumption of cereals and pieces of fruits was associated with a lower probability to belong the non-healthy nutritional status class. Future research should be deployed to distinguish between sugary and non-sugary yogurt consumption or whole grain or refined grain cereals.

In the case of the sleep behaviour, although the mean duration at the beginning of the experiment was withing the recommended range, it was also increased from 7.86 to 8.46 hours. Regarding sedentary behaviour, the percentage of children that have meals without screens increased until 55.56%.

In the physical activity domain, the IoT measured variables together with the validate tool PAQ-C scoring presented potential trends with non-healthy nutritional status: high burned calories, low number of steps, low PAQ-C scoring and low time of active minutes. Moreover, lower time of total sleep duration trend to be associated with a lower probability to belong to the non-healthy nutritional status class. It is also important to highlight that despite the COVID-19 situation, the mean number of steps increased from 5,170 to 7,176.

To assess the satisfaction and user experience of the OCARIoT solution, two standard questionnaires were used: 1) User Experience Questionnaire (UEQ-S) [43], revealed that the OCARIoT solution created a high positive impression, and the quality is excellent at overall level ( $>$90%); 2) Unified Theory of Acceptance and Use of Technology (UTAUT) [44], with very positive feedback at overall level ( $>$80%). As future work, the OCARIoT solution needs further refinement to become a commercial tool.

## IV. Conclusion

More than 300 children were involved in the Phase 1 for initial baseline collection and participation in app validation workshops. In Phase 2, almost 200 children were involved in the 4 schools from Spain, Greece and Brazil. The results of this study demonstrate the effectiveness of the OCARIoT solution improving children adherence to healthy habits and their nutritional status, decreasing the prevalence of obesity in 75.5% from baseline levels in the intervention group and decreasing the percentage of children with thinness to 1,33%. At the end of the experiment could be observed an increase in the percentages of recommended “green” consumption, mainly in fruits and vegetables, reaching almost 60% of children. Regarding sedentary behaviour, the mean number of steps and the percentage of children that have meals without screens increase despite the COVID-19 pandemic. This data collection confirms our preliminary hypothesis about the OCARIoT health model and the potential obesity risk variables associated with the BMI for age categorization.

The outcomes analysis about the usage of the solution during piloting, confirms that OCARIoT ecosystem can assess the behaviours of children, motivating and guiding them towards achieving personal behavioural goals. Although it is demonstrated that the environmental status can directly influence our health, at this stage it is still not possible to relate the data collected to the quality of health of children in relation to their physical activities due to problems related to COVID-19, as the data were collected in their absence in schools. From user experience and technology acceptance perspective, the results revealed that the OCARIoT solution created a high positive impression and satisfaction.

To promote sustainability, these interventions must be consistent with a broader effort to create a culture of positive behaviour. While it may require some additional effort from teachers and healthcare professionals to implement and sustain these interventions, the benefits can be significant in terms of reducing the need for more intensive interventions later on. By engaging families, educators and healthcare professionals, we can create a culture of collaboration that can help ensure the sustainability of the intervention over time. In addition, sharing responsibility for tracking progress and providing support may contribute alleviate the burden on individual teachers and healthcare professionals, making it more feasible to sustain the intervention in the long run. However, the OCARIoT solution needs further refinement to become a scalable and sustainable commercial tool, which has been considered for future work.
